# P-217. Risk Factors Associated with *Clostridioides difficile* Infection and Mortality in a Referral Hospital in Nicaragua

**DOI:** 10.1093/ofid/ofae631.421

**Published:** 2025-01-29

**Authors:** Carlos Balitán-Amoreti, Sunaya Marenco-Avilés, Guillermo D Porras-Cortés

**Affiliations:** Hospital Dr. Fernando Vélez Paiz, Masaya, Masaya, Nicaragua; Hospital Dr. Fernando Vélez Paiz, Masaya, Masaya, Nicaragua; Hospital Dr. Fernando Vélez Paiz, Masaya, Masaya, Nicaragua

## Abstract

**Background:**

*Clostridioides difficile* infection (CDI) is one of the main problems among healthcare-associated infections. Some studies report an increase in the incidence of this infection causing enterocolitis in hospitalized patients and eventually being associated with various complications, mortality, and increased hospital costs. The data about the epidemiology and outcomes of CDI in Nicaragua are insufficient. The aim of this study was to determine risk factors associated with CDI infection and mortality in patients admitted to a referral hospital (Dr. Fernando Vélez Paiz Hospital) in Managua, Nicaragua.

**Table 1.**
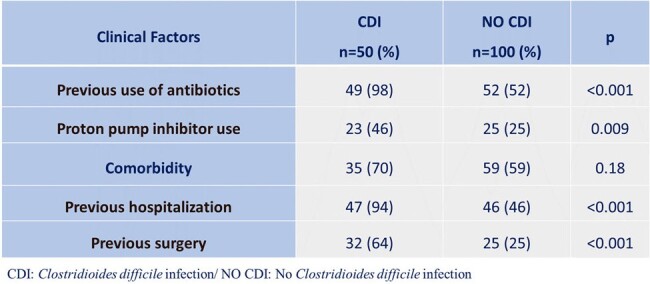
Clinical Factors Associated in Patients with and without Clostridioides difficile Infection

**Methods:**

It is an analytical nested cohort study. A total of 150 patients hospitalized in different services of the hospital between January 2020 and December 2022 and who started with in-hospital diarrhea (defined as the absence of pre-existing diarrhea prior to hospitalization and onset 72 hours after admission to the hospital) were included. Of the150 patients, 50 were defined as CDI cases and 100 as controls without CDI. The diagnosis of CDI was made by molecular method (real-time RT-PCR) using Xpert C. difficile®. Differences between cases and controls were analyzed.

**Figure 1.**
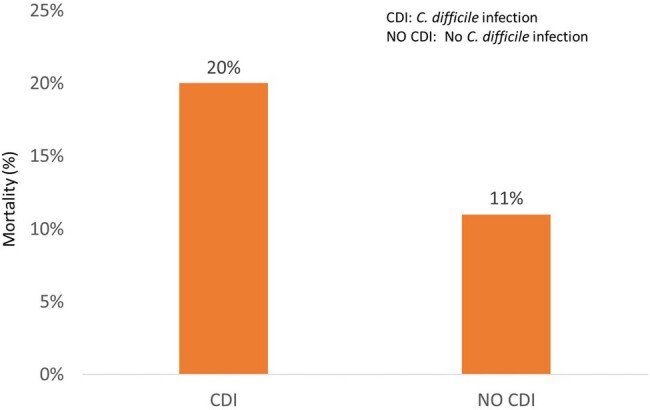
Comparative Mortality Rate in Patients with and without Clostridioides difficile Infection

**Results:**

The mean age of the patients was 52.8±18.7 years-old, 52% were men, and 50% had an associated chronic disease. A higher proportion of patients with CDI had a history of antibiotic use (98% vs 52%, p< 0.001), use of proton pump inhibitors (46% vs 25%, p=0.009), previous hospitalization (94% vs 46%, p< 0.001), and previous surgery (64% vs 25%, p< 0.001) (Table 1). Mortality in patients with CDI was 20% vs. 11% in controls (Figure 1). In the univariate analysis, the risk factors for CDI were the classic ones, such as previous use of antibiotics (ceftriaxone, quinolones, clindamycin), previous hospitalization, previous surgery, and use of proton pump inhibitors. The multivariate analysis found that previous use of ceftriaxone (OR: 6.40; 95%CI: 2.09–19.59) and clindamycin (OR: 3.0; 95%CI: 1.01–8.92) were significantly associated with CDI (Table 2). Regarding mortality, a significant association was found with age over 60 years-old (OR: 4.33; 95%CI: 1.02-18.41).

**Table 2.**
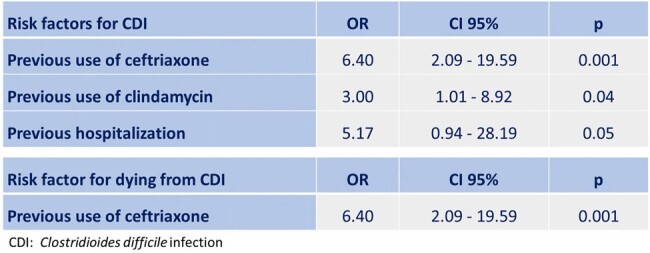
Risk Factors in Multivariate Analysis for Clostridioides difficile Infection and Mortality

**Conclusion:**

Prior use of ceftriaxone and clindamycin are independent risk factors for CDI. Patients with CDI over 60 years-old have a higher risk of mortality.

**Disclosures:**

**All Authors**: No reported disclosures

